# Efficacy and safety of esaxerenone (CS-3150), a newly available nonsteroidal mineralocorticoid receptor blocker, in hypertensive patients with primary aldosteronism

**DOI:** 10.1038/s41440-020-00570-5

**Published:** 2020-11-16

**Authors:** Fumitoshi Satoh, Sadayoshi Ito, Hiroshi Itoh, Hiromi Rakugi, Hirotaka Shibata, Atsuhiro Ichihara, Masao Omura, Katsutoshi Takahashi, Yasuyuki Okuda, Setsuko Iijima

**Affiliations:** 1 Division of Clinical Hypertension, Endocrinology and Metabolism, Tohoku University Graduate School of Medicine, 2-1 Seiryo-machi, Aoba-ku, Sendai, Miyagi 980-8575 Japan; 2 Division of Nephrology, Endocrinology and Vascular Medicine, Department of Medicine, Tohoku University School of Medicine, 2-1 Seiryo-machi, Aoba-ku, Sendai, Miyagi 980-8575 Japan; 3 Katta General Hospital, 36 Shimoharaoki, Fukuokakuramoto, Shiroishi, Miyagi 989-0231 Japan; 4 Department of Endocrinology, Metabolism and Nephrology, Department of Internal Medicine, Keio University School of Medicine, 35 Shinano-machi, Shinjuku-ku, Tokyo, 160-8582 Japan; 5 Department of Geriatric and General Medicine, Osaka University Graduate School of Medicine, 2-2 Yamadaoka, Suita, Osaka 565-0871 Japan; 6 Department of Endocrinology, Metabolism, Rheumatology and Nephrology, Faculty of Medicine, Oita University, 1-1 Idaigaoka, Hasama, Yufu, Oita 879-5593 Japan; 7 Department of Endocrinology and Hypertension, Tokyo Women’s Medical University, 8-1 Kawada-cho, Shinjuku-ku, Tokyo, 162-8666 Japan; 8 Minatomirai Medical Square, 3-6-3 Minatomirai, Nishi-ku, Yokohama, 220-0012 Japan; 9 Showa General Hospital, 8-1-1 Hanakoganei, Kodaira, Tokyo, 187-8510 Japan; 10 Daiichi Sankyo Co., Ltd, 1-2-58 Hiromachi, Shinagawa-ku, Tokyo, 140-8710 Japan

**Keywords:** Esaxerenone, Primary aldosteronism, Hypertension, Renin, Aldosterone

## Abstract

Mineralocorticoid receptor (MR) blockers are very beneficial for patients with hypertension and primary aldosteronism (PA). We investigated the efficacy and safety of a newly available nonsteroidal MR blocker, esaxerenone, in Japanese patients with hypertension and PA. A multicenter, open-label study was conducted in Japan between October 2016 and July 2017. Patients with hypertension and PA received 12 weeks of treatment with esaxerenone, initiated at 2.5 mg/day and escalated to 5 mg/day during week 2 or 4 of treatment, based on individual response. The only other permitted antihypertensive therapies were stable dosages of a Ca^2+^ channel blocker or α-blocker. The primary efficacy outcome was a change in sitting systolic and diastolic blood pressure (SBP/DBP) from baseline to the end of treatment. Forty-four patients were included; dose escalation to 5 mg/day was implemented for 41 of these patients. Significant decreases in SBP and DBP were observed (point estimates [95% confidence interval] −17.7 [−20.6, −14.7] and −9.5 [−11.7, −7.3] mmHg, respectively; both *p* < 0.0001 at the end of treatment). Significant BP reductions were evident from week 2 and continued through to week 8; BP remained stable until week 12. The antihypertensive effect of esaxerenone on SBP was significantly greater in females and in patients receiving monotherapy. The major drug-related adverse events were serum K^+^ increase and estimated glomerular filtration rate decrease (both 4.5%, *n* = 2); no gynecomastia or breast pain was observed. We conclude that esaxerenone is a potent MR blocker with favorable efficacy and safety profiles in patients with hypertension and PA.

## Introduction

Primary aldosteronism (PA) is a well-recognized form of secondary hypertension, and ~5–9% of all patients with hypertension have PA [1]. The prevalence of PA is ~20% in those with treatment-resistant hypertension [2, 3].

Compared with patients with essential hypertension, patients with PA have an independent association with higher risks of cerebro- and cardiovascular complications (e.g., stroke, cardiac hypertrophy, atrial fibrillation, coronary artery disease, and cardiac failure), metabolic disturbances, and renal insufficiency [4–9].

Patients with unilateral PA can be successfully treated with surgical adrenalectomy, but pharmacologic therapy is suggested for those who do not want surgery or for those with bilateral PA [10, 11]. Mineralocorticoid receptor (MR) blockers are the principal agents shown to be beneficial in patients with PA [12, 13], and a systematic review demonstrated the superiority of MR blockers over adrenalectomy [14]. The benefits of add-on therapy with an MR blocker in patients with resistant hypertension have also been documented in several studies [15–18]. These findings are reflected in PA clinical practice guidelines that recommend treatment with an MR blocker to manage hypertension and prevent cardiovascular disorders and target organ damage [10, 19].

Currently available MR blockers include spironolactone and eplerenone [20], both of which have antihypertensive effects in patients with PA [12, 13]. However, the clinical use of these agents is limited by problematic adverse effects. For example, spironolactone has low MR-binding specificity and therefore is associated with sex hormone receptor-related adverse events [21–23]. The use of either agent is complicated by hyperkalemia [20, 23, 24], and eplerenone is contraindicated in patients with type 2 diabetes mellitus and albuminuria or proteinuria and in those with renal impairment (creatinine clearance <50 mL/min) [24, 25]. Eplerenone is also contraindicated in combination with K^+^ supplements [20]. Hence, there is currently an unmet clinical need for an MR blocker with an improved safety profile that can be administered to a wide range of patients, including those with comorbidities.

Esaxerenone is an oral nonsteroidal MR blocker with high MR-binding specificity [26]. Data from clinical studies [27–29] have shown that esaxerenone inhibits MR activity, is well tolerated and reduces BP in a dose-dependent manner in Japanese patients with essential hypertension. The phase 3 study [29] also showed that esaxerenone 2.5 mg/day was noninferior to eplerenone 50 mg/day and that esaxerenone at 5 mg/day exhibited superior BP-lowering effects over esaxerenone at 2.5 mg/day. In a study of esaxerenone in patients with moderate renal dysfunction with diabetes with albuminuria [30], incremental dose escalation starting from a low dose was able to manage serum K^+^ elevations and renal function safely while exerting antihypertensive effects. Data from these studies provide important guidance regarding the role of esaxerenone in the management of hypertension with related comorbidities.

The current clinical study investigated the efficacy and safety of esaxerenone in patients with hypertension and PA.

## Materials and methods

### Study design

This multicenter, open-label, 12-week study (NCT02885662; JapicCTI-163349) was conducted in Japan between October 2016 and July 2017 (Supplementary Fig. 1). The study protocol was approved by the institutional review board at each study center, and all patients provided written informed consent prior to enrollment. All study procedures were conducted in accordance with Good Clinical Practice, applicable local regulations, and the ethical principles of the Declaration of Helsinki.

### Patients

The patients were aged ≥20 years and diagnosed with PA within the previous 5 years based on a screening test (plasma aldosterone concentrations [PAC] >120 pg/mL and aldosterone-to-renin ratio [ARR] >200) and at least one positive confirmatory PA test result from among the following: captopril test (ARR >200 at 60 or 90 min after loading captopril 50 mg); physiological saline challenge test (PAC >60 pg/mL 4 h after loading saline 0.9%); furosemide standing test (plasma renin activity [PRA] <2.0 ng/mL/h 2 h after loading furosemide 40 mg); or oral salt loading test (24-h urinary aldosterone >8 μg/day, with urinary sodium >170 mEq/day). All tests to confirm a positive diagnosis of PA were conducted in accordance with the Japanese Endocrine Society guidelines [31].

Additional inclusion criteria, as per Japanese Society of Hypertension Guidelines [19], included stable sitting systolic BP (SBP) ≥140 and <180 mmHg and stable sitting diastolic BP (DBP) ≥90 and <110 mmHg at the last two assessments during the observation period. Eligible patients had not taken antihypertensives for at least 4 weeks prior to the start of the study or had been taking a stable dosage of only one basic antihypertensive agent (a Ca^2+^ channel blocker [CCB], except for cilnidipine, efonidipine, azelnidipine, and benidipine, or an α-blocker, which have reduced effects on the secretion of renin and aldosterone) before and during the treatment period.

Patients with other forms of secondary hypertension (e.g., renovascular hypertension, Cushing’s syndrome, subclinical Cushing’s syndrome, pheochromocytoma, or hypertension associated with a single kidney) or hypertensive crisis were excluded. Other exclusion criteria included type 1 diabetes mellitus, diabetic nephropathy, serious hepatic disease, a history of stroke or transient ischemic attack, orthostatic hypotension, cerebral or cardiovascular diseases, adrenal surgery or revascularization of the carotid or peripheral arteries in the previous 6 months, hospitalization for severe hyperkalemia within the previous year, history of adverse drug reactions to eplerenone or spironolactone, and serum K^+^ level <3.0 or ≥5.1 mEq/L or an estimated glomerular filtration rate (eGFR) <30 mL/min/1.73 m^2^. Prohibited concomitant medications included antihypertensive agents (other than the aforementioned permitted concomitant medications), diuretics, other sympathetic blocking agents, vasodilators, and renin inhibitors, though the use of short-acting oral nitrates was permitted in an emergency.

### Study treatments and administration

After determination of eligibility and provision of informed consent, primary enrollment was performed. The duration of the pretreatment observation period was determined by previous therapies. The washout period was 9 weeks for diuretics and 4 weeks for any other antihypertensive medication. As noted above, the use of one antihypertensive agent (CCB or α-blocker) was permitted during the observation period if the dosage remained unchanged for at least 4 weeks prior to initiation of the study treatment.

The observation period was followed by secondary enrollment (treatment initiation) and a 12-week treatment period. The dosage of esaxerenone was based on the results of a previous phase 2 clinical study [28]. Esaxerenone in the present study was initiated at a dosage of 2.5 mg/day and escalated to 5 mg/day during week 2 or 4 of treatment if SBP/DBP was ≥120/≥80 mmHg, serum K^+^ level was <5.1 mEq/L, and decreases in eGFR from baseline were <30%. There was a 1-week post treatment follow-up period after the completion of the esaxerenone therapy, during which follow-up tests and examinations were conducted.

### Study endpoints

The primary efficacy outcome was a change in sitting SBP and DBP from baseline to the end of esaxerenone treatment. Secondary efficacy endpoints included changes in sitting BP (SBP/DBP) over time and the percentage of patients achieving the target sitting BP (Target 1: BP of <140/90 mmHg among all patients, Target 2: BP of <130/80 mmHg among patients with type 2 diabetes mellitus and <140/90 mmHg among those without type 2 diabetes mellitus). Safety endpoints included adverse events, laboratory test values, and vital signs. Additionally, the proportions of patients with serum K^+^ levels ≥5.5 mEq/L, ≥6.0 mEq/L, or ≥5.5 mEq/L on two consecutive occasions were determined. Pharmacodynamic assessments included time-course and change from baseline in PAC, PRA, and ARR.

### Assessments

On each occasion, three sitting BP measurements were taken at 1- to 2-min intervals in an office after a resting period of a least 5 min, and the mean value was recorded as the BP at that visit. All measurements were taken within 21–27 h of study drug administration, and the measurer and measuring device (automatic blood pressure monitor) were the same for each patient. The patients were questioned about the occurrence of adverse events at each study visit. PAC was determined using a radioimmunoassay and PRA using an enzyme immunoassay [27].

### Statistical analysis

The target sample size was set at 40 patients based on feasibility; this was confirmed to be sufficient to detect a statistically significant change from baseline in sitting BP (SBP/DBP) assuming a true change of −10/−5 mmHg with SD of 15/10 mmHg. Patients who received at least one dose of study drug and had at least one efficacy variable measured were included in the full analysis set (FAS), and all patients who provided informed consent and received at least one dose of study drug were included in the safety analysis set (SAS). Point estimates and corresponding 95% confidence interval (CI) values for the difference in sitting BP between baseline and the end of treatment were calculated and analyzed using a paired *t*-test. The method of last observation carried forward was used to impute missing BP data.

The geometric mean percent change (and 95% CI) in PAC and PRA from baseline to week 12 was calculated using log-transformed values. Missing PRA values, due to the limit of quantification of the assay, were assigned using the lower limit of quantification value (0.2 ng/mL/h).

Subgroup analyses were conducted based on sex, age (<65 vs ≥65 years), body mass index (BMI; <25 vs ≥25 kg/m^2^), baseline SBP (<160 vs ≥160 mmHg), baseline DBP (<100 vs ≥100 mmHg), prior use of antihypertensive agents, concomitant antihypertensive agents, ARR (above and below the median), diabetes mellitus (yes vs no), PA subtype (including unilateral adrenal lesion, bilateral adrenal lesions, and disease classification of either an aldosterone-producing tumor or idiopathic hyperaldosteronism (IHA)), and use of K^+^ supplements. Safety variables are summarized in a descriptive manner.

Post hoc analyses consisted of the following: the statistical significance of changes in BP, eGFR, and serum K^+^ level over time, as assessed using paired *t-*tests; the exploratory subgroup analysis by PRA at the end of the treatment (<1 vs ≥1 ng/mL/h); and the statistical comparisons among each patient subgroup.

Categorical variables are presented as absolute values and percentages and continuous variables as the mean ± SD, unless otherwise specified. All reported *p* values are two-sided; those <0.05 are considered to be statistically significant (without adjustment for multiple testing). All statistical analyses were performed using SAS System Release 9.3 (SAS Institute Inc., Cary, NC, USA).

## Results

### Patient demographics

A total of 44 patients were enrolled in the study (mean age 49.6 ± 9.68 years, 57% female) (Table 1). The majority of patients had bilateral adrenal lesions and IHA (79.5%) and had received prior antihypertensive therapy (86.4%). In those with previous antihypertensive treatment, the most commonly used agents were CCBs (72.7%); CCBs were also the most commonly used concomitant antihypertensive therapy (65.9%). Of the 44 enrolled patients, 41 completed the study (two withdrew due to adverse events, and one withdrew consent). All 44 patients were included in the FAS and SAS.

**Table 1:** Patient characteristics

Characteristics	Patients (*n* = 44)
Age, years	49.6 ± 9.68
Female, *n* (%)	25 (56.8)
Weight, kg	65.3 ± 13.2
Body mass index, kg/m^2^	24.5 ± 3.3
Localization/PA subtype, *n* (%)	
Unilateral adrenal lesion/aldosterone-producing adenoma	4 (9.1)
Bilateral adrenal lesion/idiopathic hyperaldosteronism	35 (79.5)
Bilateral adrenal lesion/unknown	3 (6.8)
Familial hyperaldosteronism	1 (2.3)
Unknown	1 (2.3)
SBP, mmHg	154.0 ± 9.8
DBP, mmHg	100.0 ± 5.9
eGFR, mL/min/1.73 m^2^	78.5 ± 13.8
<60 mL/min/1.73 m^2^, *n* (%)	2 (4.5)
Serum K^+^, mEq/L	4.01 ± 0.33
<3.5 mEq/L, *n* (%)	3 (6.8)
Diabetes mellitus, *n* (%)	5 (11.4)
LDL-C, mg/dL	127.9 ± 27.7
PAC, pg/mL	229.8 ± 396.7
PRA, ng/mL/h	0.49 ± 0.51
ARR	829.9 ± 2036.6
Urinary Na^+^/K^+^ ratio	2.3 ± 1.5
Prior antihypertensive treatment^a^, *n* (%)	38 (86.4)
MR blocker	9 (20.5)
Ca^2+^ channel blocker	32 (72.7)
α-blocker	1 (2.3)
β-blocker	2 (4.5)
Concomitant antihypertensive agent, *n* (%)	30 (68.2)
Ca^2+^ channel blocker	29 (65.9)
α-blocker	1 (2.3)
K^+^ supplements, *n* (%)	6 (13.6)

Values are means ± standard deviations, or numbers of patients (%)

*ARR* aldosterone-renin ratio, *LDL-C* low-density lipoprotein cholesterol, *MR* mineralocorticoid receptor, *PA* primary aldosteronism, *PAC* plasma aldosterone concentration, *PRA* plasma renin activity

^a^In the 4 weeks before screening

### Efficacy

In 41 of the 44 patients, the esaxerenone dosage was increased to 5 mg/day. In the other three, the dose remained at 2.5 mg/day. Of these, the dosage was not increased due to a hypotensive effect in two patients, and the remaining one patient discontinued the study treatment prior to the dosage increase visit. Of the three patients who withdrew from the study, esaxerenone dosages were titrated to 5 mg/day in two, whereas one remained on esaxerenone 2.5 mg/day.

**Fig. 1:**
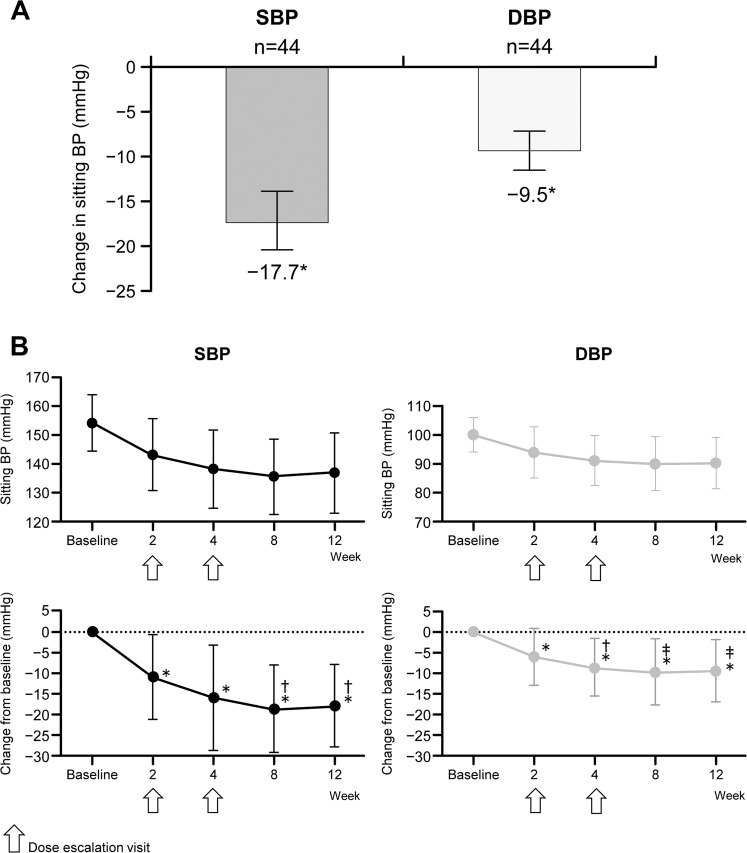
Overall change (means ± SDs) from baseline (**A**) and change over time (**B**) in sitting systolic blood pressure (SBP) and diastolic blood pressure (DBP) (full analysis set). *p < 0.0001 vs baseline (paired *t*-test); ^†^p < 0.05 vs BP at week 2; ^‡^*p* < 0.01 vs BP at week 2 (paired *t*-test)

Mean ± SD sitting SBP values were 154.0 ± 9.8 mmHg at baseline and 136.4 ± 13.6 mmHg at the end of treatment; corresponding values for DBP were 100.0 ± 5.9 and 90.5 ± 8.6 mmHg. Significant decreases in SBP and DBP were observed between baseline and the end of the esaxerenone treatment period; point estimates (95% CI) for the changes in sitting SBP and DBP from baseline to the end of the treatment were −17.7 (−20.6, −14.7) mmHg and −9.5 (−11.7, −7.3) mmHg, respectively (both *p* < 0.0001) (Fig. 1a). Significant decreases from baseline in sitting BP were evident from week 2 of treatment; BP continued to decrease through week 8 and remained stable until week 12 (Fig. 1b). In the analysis of final esaxerenone dosage, changes from baseline to the end of treatment in SBP and DBP were −20.7 and −15.3 mmHg with 2.5 mg/day (*n* = 3) and −17.4 and −9.1 mmHg with 5 mg/day (*n* = 41), respectively.

Among all patients, the percentage (95% CI) of patients who achieved a sitting BP of <140/90 mmHg at the end of treatment (Target 1) was 47.7% (32.5, 63.3). The percentage (95% CI) of patients who achieved a sitting BP of <130/80 mmHg among those with type 2 diabetes and <140/90 mmHg among those without diabetes was 40.9% (26.3, 56.8) at the end of treatment (Target 2).

**Fig. 2:**
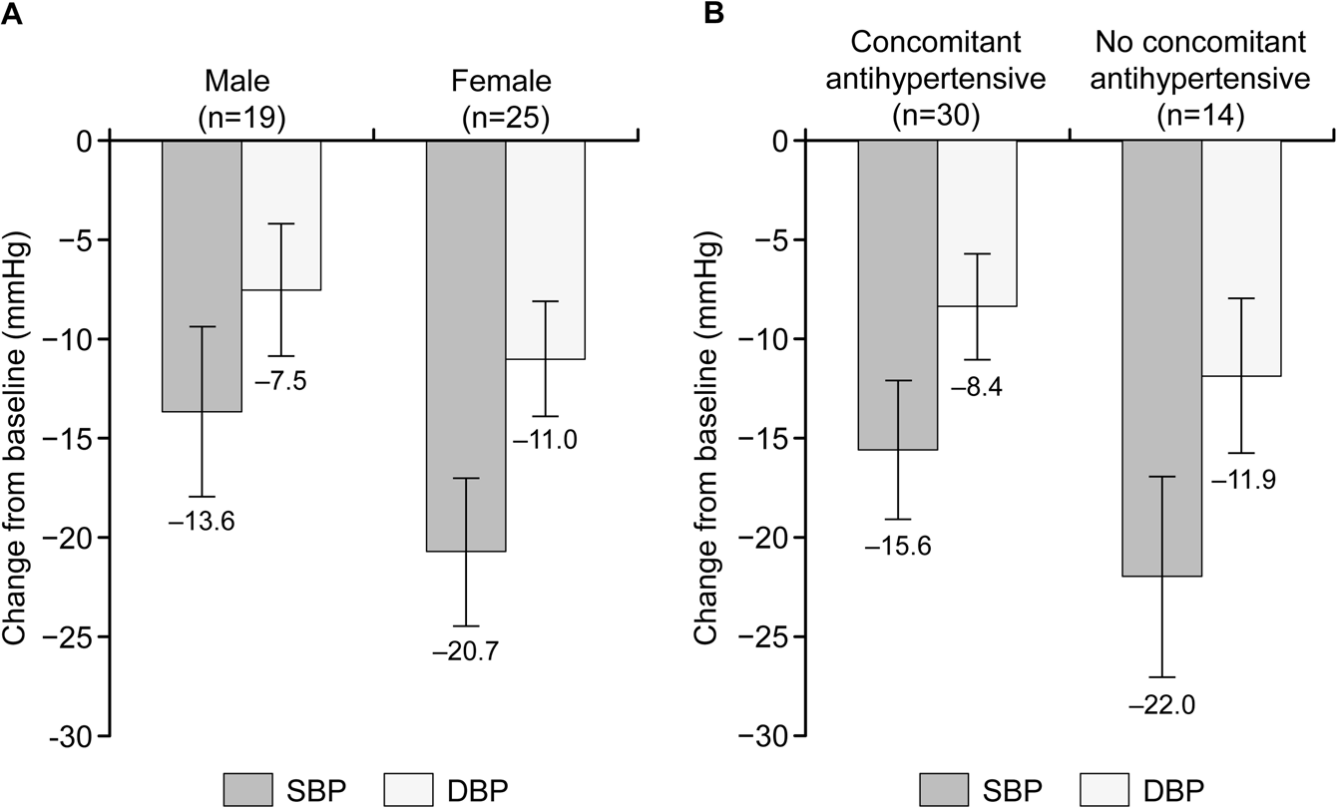
Differences (means ± 95% confidence intervals) in systolic blood pressure (SBP) and diastolic blood pressure (DBP) from baseline to the end of treatment according to sex (**A**) and administration of concomitant antihypertensive agents (**B**). Significant differences in SBP reduction were observed in the following comparisons: females vs males (*p* = 0.0156) and without vs with concomitant antihypertensive agents (*p* = 0.0433)

In general, consistent antihypertensive effects were observed, but numerically different trends were observed in some subgroups (unilateral or bilateral adrenal lesions, PRA at the end of the treatment <1 or ≥1) (Supplementary Table 1). When differences between groups were examined by sex and by use of concomitant antihypertensives, significant differences in SBP reduction were observed between females and males (*p* = 0.0156; Fig. 2a) and between patients not receiving concomitant antihypertensive agents and those who received concomitant antihypertensive agents (*p* = 0.0433; Fig. 2b). Fourteen patients did not receive any concomitant antihypertensives, and changes from baseline to the end of treatment in SBP and DBP were −22.0 and −11.9 mmHg, respectively, in the subgroup receiving esaxerenone monotherapy and −15.6 and −8.4 mmHg, respectively, in the subgroup receiving esaxerenone with concomitant antihypertensives (Supplementary Table 2). In subgroup analysis by PRA at the end of the treatment, changes from baseline to the end of treatment in SBP and DBP were −20.0 and −10.9 mmHg, respectively, in patients with PRA ≥1 ng/mL/h at week 12 (*n* = 17) and −16.5 and −8.7 mmHg, respectively, in patients with PRA <1 ng/mL/h (*n* = 24) (Supplementary Table 1).

### Plasma aldosterone and renin activity

Both PAC and PRA increased from baseline by 83.9 pg/mL and 0.60 ng/mL/h, respectively, after 12 weeks of treatment with esaxerenone. Based on percent change, both of these values were statistically significant (*p* < 0.001, Supplementary Fig. 2).

### Safety

The overall rate of treatment-emergent adverse events was 61.4% (27/44), the most common being dizziness (9.1%) (Table 2). One female patient reported menorrhagia during treatment with esaxerenone. Adverse events considered to be related to the study treatment were reported in 25.0% of patients (11/44); increased serum K^+^ levels and decreased eGFR each occurred in two patients (4.5%) (Table 2). One serious adverse event of moderate severity occurred in one patient (bronchitis), though it was determined to be unrelated to esaxerenone. Two patients discontinued esaxerenone treatment due to adverse events, one due to a serious adverse event (bronchitis, described above) and the other due to increased serum K^+^ levels. No deaths occurred during the study.

**Table 2:** Adverse events (*n* ≥ 2) and major drug-related adverse events

Category	Patients (*n* = 44)
Any adverse events	27 (61.4)
Dizziness	4 (9.1)
Viral upper respiratory tract infection	3 (6.8)
Bronchitis	2 (4.5)
Cystitis	2 (4.5)
Pharyngitis	2 (4.5)
Abdomen discomfort	2 (4.5)
Increase in serum K^+^	2 (4.5)
Increase in γ-glutamyl transferase	2 (4.5)
Reduction in eGFR	2 (4.5)
Drug-related adverse events	11 (25.0)
Increase in serum K^+^	2 (4.5)
Reduction in eGFR	2 (4.5)

Values are *n* (%)

*eGFR* estimated glomerular filtration rate

**Fig. 3:**
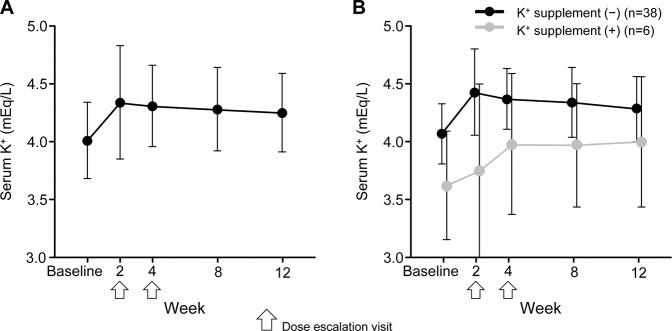
Change (means ± SDs) in serum K^+^ levels over time overall (**A**) and in the presence or absence of concomitant K^+^ supplementation (**B**)

**Fig. 4:**
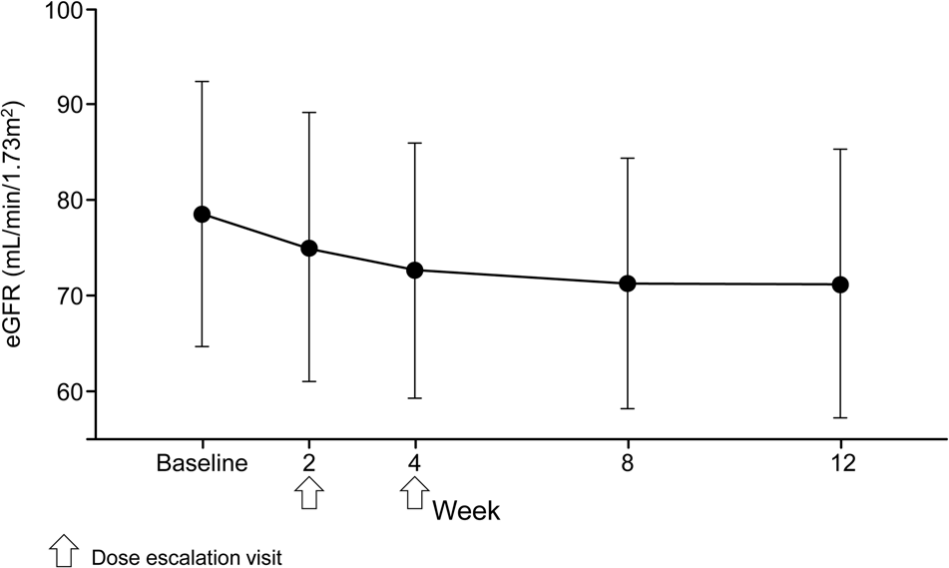
Change (means ± SDs) from baseline in estimated glomerular filtration rate (eGFR) (safety analysis set)

Mean ± SD serum K^+^ levels increased from 4.01 ± 0.331 mEq/L at baseline to 4.34 ± 0.491 mEq/L at week 2, but serum K^+^ levels did not continually increase throughout the treatment period even after dose titration at week 2 or 4 (Fig. 3a). A serum K^+^ level of ≥6.0 mEq/L at 2 weeks after initiation of therapy in one patient during treatment with esaxerenone 2.5 mg/day was reported as an adverse event. Esaxerenone was discontinued in this patient, and serum K^+^ decreased to 5.2 mEq/L 5 days later. Changes in serum K^+^ levels showed similar trajectories between patients who were and were not receiving concomitant therapy with K^+^ supplements (Fig. 3b). The eGFR decreased by 8.1 mL/min/1.73 m^2^ at week 8 compared with baseline, and this decrease was maintained to week 12 (Fig. 4). There were no marked changes in any other clinical laboratory test results, vital signs, or ECG findings during esaxerenone treatment.

## Discussion

This study is the first to investigate the antihypertensive effects of a novel MR blocker, esaxerenone, in hypertensive patients diagnosed with PA. Esaxerenone had a clinically significant antihypertensive effect in Japanese hypertensive patients with PA and was well tolerated, with consistent efficacy across a range of patient subgroups. Additionally, dose escalation from esaxerenone 2.5–5 mg/day was feasible and safe; this was achieved in 41/44 (93%) of the patients of whom 37 (90%) underwent dose titration at week 2. Significant decreases from baseline in sitting BP were evident from week 2 of treatment, and additional significant reductions were observed at week 8 compared with week 2, indicative of the effects of dose escalation to 5 mg.

Medical therapy including an MR blocker is recommended as first-line treatment in patients with bilateral PA (IHA) and those with unilateral PA (aldosterone-producing adenoma [APA]) who are unable or unwilling to undergo surgery [10, 31]. Although the number of APA patients in this study was small, significant BP-lowering effects of esaxerenone were observed in both APA and IHA.

Of note, the results of our study indicate that the antihypertensive effect of esaxerenone on SBP was significantly greater in female patients and in patients without concomitant use of other antihypertensive agents, even though the number of patients was small. These data are in line with previous data from an international consensus study investigating surgical treatment of unilateral PA [32]. In that analysis, female patients were found to have a higher likelihood of complete clinical success, defined as normal blood pressure without the aid of antihypertensive medication, than male patients (odds ratio 2.25, 95% CI 1.40, 3.62; *p* = 0.001). Furthermore, patients with fewer antihypertensive medications were more likely to achieve complete clinical success (*p* < 0.001) [32]. Female hormones are known to have vasoprotective effects, and differences in antihypertensive effect based on sex are likely driven by the vasoprotective role of estrogen in premenopausal women, which may be able to reduce irreversible vascular damage [32]. In addition, BP may be well controlled in these patients, and the duration of PA may be relatively short [32].

The reductions in SBP and DBP achieved with esaxerenone in PA in the current study (−17.7 and −9.5 mmHg, respectively) are clinically relevant; the effects are not less than those achieved with eplerenone (100–300 mg/day) in patients with hypertension and PA in a previous study but are smaller than those associated with spironolactone (75–225 mg/day) treatment [13]. However, in that study, both MR blockers were administered as monotherapy at dosages higher than the approved doses, and the study design and study population differed markedly from those in our analysis. In our study, SBP and DBP reductions were −22.0 and −11.9 mmHg, respectively, in the subgroup of 14 patients receiving esaxerenone as monotherapy, which was almost comparable to the effect of spironolactone 150 mg/day at week 8 in the prior study [13]. Regardless, we cannot yet draw any definite conclusions regarding the antihypertensive effect of esaxerenone compared with these existing MR antagonists.

In previous studies, sex hormone-related side effects (particularly gynecomastia and breast pain) were significantly more common in patients treated with spironolactone compared with eplerenone, and the rate of hyperkalemia was also highest with spironolactone [12, 13]. In the present study, no gynecomastia or breast pain was reported; however, one female patient did report menorrhagia during treatment with esaxerenone 5 mg/day. In clinical studies of esaxerenone [28, 29], no sex hormone receptor-related AEs or menorrhagia were observed in any patients treated with esaxerenone.

Although serum K^+^ levels are normal in most patients, up to 40% of Japanese patients with PA have hypokalemia [10, 33]. The mechanism of hypokalemia in PA occurs via stimulation of MRs by excessive aldosterone, which promotes Na^+^ reabsorption and K^+^ excretion and contributes to the development of hypertension. The PA patients in the current study had a mean baseline serum K^+^ level of 4.01 mEq/L, and three patients (6.8%) had hypokalemia (serum K^+^ <3.5 mEq/L) at initiation of the study treatment despite taking K^+^ supplements. As a result of esaxerenone treatment, serum K^+^ levels increased to ≥3.5 mEq/L in two of the three patients at week 12. Elevated serum K^+^ to ≥5.5 mEq/L was observed in one patient during esaxerenone therapy, and the overall incidence was within a clinically acceptable range. In contrast, both spironolactone and eplerenone have been associated with the development of hyperkalemia when used to treat PA [34], and the use of K^+^ supplements is contraindicated during treatment with eplerenone. Our study protocol allowed the use of concomitant K^+^ supplements; six patients received K^+^ supplements at the initiation of the study, no hyperkalemia was observed, and increases in serum K^+^ levels were within a preferable range in all patients.

Glomerular hyperfiltration is a predictor of excessive aldosterone-related renal damage [35]. In patients with PA, renal glomerular hyperfiltration is present due to an excess of aldosterone, which causes elevation of eGFR. These elevations appear to be reduced by treatment with an MR blocker due to improved glomerular hypertension [36, 37]. In our study, the extent of reductions in eGFR during treatment of PA with esaxerenone was similar to that seen with other MR blockers, indicating that correction of hyperfiltration probably contributed to the observed reduction in eGFR, which may indicate recovery of the tubuloglomerular feedback damaged by hyperaldosteronism [38]. Given that eGFR tended to return to baseline levels after the end of esaxerenone treatment in other studies [30, 39], the eGFR reductions in this study are considered to be caused by hemodynamic changes rather than organic renal damage.

## Limitations

One important limitation of this study is the relatively short duration of treatment. Other features of the study design, including the small sample size, lack of randomization to treatment, and absence of a placebo or comparator arm, are also limitations. Nonetheless, this study contributes to a wider comprehensive clinical development program covering multiple aspects of esaxerenone therapy.

## Conclusions

The results of this open-label phase 3 study suggest that esaxerenone has favorable efficacy and safety profiles in hypertensive patients with unilateral or bilateral PA. Dose escalation was successful in the majority of patients, and esaxerenone was well tolerated. Guidelines recommend an MR blocker as first-line therapy in PA patients with bilateral adrenal lesions and in unilateral adrenal lesion patients unable or unwilling to undergo surgery. Therefore, esaxerenone may be widely used in PA patients regardless of PA subtype and may have important applications in those whose tumor lesions cannot be definitively diagnosed or in settings in which facilities for definitive diagnosis are lacking. Esaxerenone is also likely to be useful in patients with treatment-resistant hypertension, of whom ~20% are suspected to have PA and in whom diagnosis confirmation is difficult.

## Supplementary information

Supplementary material

## References

[1] Rossi GP, Bernini G, Caliumi C, Desideri G, Fabris B, Ferri C (2006). A prospective study of the prevalence of primary aldosteronism in 1,125 hypertensive patients. J Am Coll Cardiol.

[2] Calhoun DA, Nishizaka MK, Zaman MA, Thakkar RB, Weissmann P (2002). Hyperaldosteronism among black and white subjects with resistant hypertension. Hypertension.

[3] Strauch B, Zelinka T, Hampf M, Bernhardt R, Widimsky J (2003). Prevalence of primary hyperaldosteronism in moderate to severe hypertension in the Central Europe region. J Hum Hypertens.

[4] Fallo F, Veglio F, Bertello C, Sonino N, Della Mea P, Ermani M (2006). Prevalence and characteristics of the metabolic syndrome in primary aldosteronism. J Clin Endocrinol Metab.

[5] Mulatero P, Monticone S, Bertello C, Viola A, Tizzani D, Iannaccone A (2013). Long-term cardio- and cerebrovascular events in patients with primary aldosteronism. J Clin Endocrinol Metab.

[6] Savard S, Amar L, Plouin PF, Steichen O (2013). Cardiovascular complications associated with primary aldosteronism: a controlled cross-sectional study. Hypertension.

[7] Born-Frontsberg E, Reincke M, Rump LC, Hahner S, Diederich S, Lorenz R (2009). Cardiovascular and cerebrovascular comorbidities of hypokalemic and normokalemic primary aldosteronism: results of the German Conn’s Registry. J Clin Endocrinol Metab.

[8] Ohno Y, Sone M, Inagaki N, Yamasaki T, Ogawa O, Takeda Y (2018). Prevalence of cardiovascular disease and its risk factors in primary aldosteronism: a multicenter study in Japan. Hypertension.

[9] Kawashima A, Sone M, Inagaki N, Takeda Y, Itoh H, Kurihara I (2019). Renal impairment is closely associated with plasma aldosterone concentration in patients with primary aldosteronism. Eur J Endocrinol.

[10] Funder JW, Carey RM, Mantero F, Murad MH, Reincke M, Shibata H (2016). The management of primary aldosteronism: case detection, diagnosis, and treatment: an Endocrine Society Clinical Practice Guideline. J Clin Endocrinol Metab.

[11] Vaidya A, Malchoff CD, Auchus RJ, AACE Adrenal Scientific Committee. (2017). An individualized approach to the evaluation and management of primary aldosteronism. Endocr Pract.

[12] Karashima S, Yoneda T, Kometani M, Ohe M, Mori S, Sawamura T (2016). Comparison of eplerenone and spironolactone for the treatment of primary aldosteronism. Hypertens Res.

[13] Parthasarathy HK, Ménard J, White WB, Young WF, Williams GH, Williams B (2011). A double-blind, randomized study comparing the antihypertensive effect of eplerenone and spironolactone in patients with hypertension and evidence of primary aldosteronism. J Hypertens.

[14] Satoh M, Maruhashi T, Yoshida Y, Shibata H (2019). Systematic review of the clinical outcomes of mineralocorticoid receptor antagonist treatment versus adrenalectomy in patients with primary aldosteronism. Hypertens Res.

[15] Williams B, MacDonald TM, Morant S, Webb DJ, Sever P, McInnes G (2015). Spironolactone versus placebo, bisoprolol, and doxazosin to determine the optimal treatment for drug-resistant hypertension (PATHWAY-2): A randomised, double-blind, crossover trial. Lancet.

[16] Chapman N, Dobson J, Wilson S, Dahlöf B, Sever PS, Wedel H (2007). Anglo-Scandinavian Cardiac Outcome Trial Investigators. Effect of spironolactone on blood pressure in subjects with resistant hypertension. Hypertension.

[17] Nishizaka MK, Zaman MA, Calhoun DA (2003). Efficacy of low-dose spironolactone in subjects with resistant hypertension. Am J Hypertens.

[18] Václavík J, Sedlák R, Plachý M, Navrátil K, Plášek J, Jarkovský J (2011). Addition of spironolactone in patients with resistant arterial hypertension (ASPIRANT): a randomized, double-blind, placebo-controlled trial. Hypertension.

[19] Shimamoto K, Ando K, Fujita T, Hasebe N, Higaki J, Horiuchi M (2014). The Japanese Society of Hypertension guidelines for the management of hypertension (JSH 2014). Hypertens Res.

[20] Pitt B, Remme W, Zannad F, Neaton J, Martinez F, Roniker B (2003). Eplerenone, a selective aldosterone blocker, in patients with left ventricular dysfunction after myocardial infarction. N Engl J Med.

[21] Colussi G, Catena C, Sechi LA (2013). Spironolactone, eplerenone and the new aldosterone blockers in endocrine and primary hypertension. J Hypertens.

[22] Sato A (2013). Mineralocorticoid receptor antagonists: their use and differentiation in Japan. Hypertens Res.

[23] Lainscak M, Pelliccia F, Rosano G, Vitale C, Schiariti M, Greco C (2015). Safety profile of mineralocorticoid receptor antagonists: Spironolactone and eplerenone. Int J Cardiol.

[24] Pelliccia F, Patti G, Rosano G, Greco C, Gaudio C (2014). Efficacy and safety of eplerenone in the management of mild to moderate arterial hypertension: systematic review and meta-analysis. Int J Cardiol.

[25] Roush GC, Ernst ME, Kostis JB, Yeasmin S, Sica DA (2016). Dose doubling, relative potency, and dose equivalence of potassium-sparing diuretics affecting blood pressure and serum potassium: systematic review and meta-analyses. J Hypertens.

[26] Arai K, Tsuruoka H, Homma T (2015). CS-3150, a novel non-steroidal mineralocorticoid receptor antagonist, prevents hypertension and cardiorenal injury in Dahl salt-sensitive hypertensive rats. Eur J Pharmacol.

[27] Kato M, Furuie H, Shimizu T, Miyazaki A, Kobayashi F, Ishizuka H (2018). Single- and multiple-dose escalation study to assess pharmacokinetics, pharmacodynamics and safety of oral esaxerenone in healthy Japanese subjects. Br J Clin Pharmacol.

[28] Ito S, Itoh H, Rakugi H, Okuda Y, Yamakawa S (2019). Efficacy and safety of esaxerenone (CS-3150) for the treatment of essential hypertension: a phase 2 randomized, placebo-controlled, double-blind study. J Hum Hypertens.

[29] Ito S, Itoh H, Rakugi H, Okuda Y, Yoshimura M, Yamakawa S (2020). Double-blind randomized phase 3 study comparing esaxerenone (CS-3150) and eplerenone in patients with essential hypertension (ESAX-HTN study). Hypertension.

[30] Itoh H, Ito S, Rakugi H, Okuda Y, Nishioka S (2019). Efficacy and safety of dosage-escalation of low-dosage esaxerenone added to a RAS inhibitor in hypertensive patients with type 2 diabetes and albuminuria: a single-arm, open-label study. Hypertens Res.

[31] Nishikawa T, Omura M, Satoh F, Shibata H, Takahashi K, Tamura N (2011). Guidelines for the diagnosis and treatment of primary aldosteronism-the Japan Endocrine Society 2009. Endocr J.

[32] Williams TA, Lenders JWM, Mulatero P, Burrello J, Rottenkolber M, Adolf C (2017). Outcomes after adrenalectomy for unilateral primary aldosteronism: an international consensus on outcome measures and analysis of remission rates in an international cohort. Lancet Diabetes Endocrinol.

[33] Umakoshi H, Tsuiki M, Takeda Y, Kurihara I, Itoh H, Katabami T (2018). Significance of computed tomography and serum potassium in predicting subtype diagnosis of primary aldosteronism. J Clin Endocrinol Metab.

[34] Karagiannis A, Tziomalos K, Papageorgiou A, Kakafika AI, Pagourelias ED, Anagnostis P (2008). Spironolactone versus eplerenone for the treatment of idiopathic hyperaldosteronism. Expert Opin Pharmacother.

[35] Schmieder RE, Messerli FH, Garavaglia G, Nunez B (1990). Glomerular hyperfiltration indicates early target organ damage in essential hypertension. JAMA.

[36] Ribstein J, Du Cailar G, Fesler P, Mimran A (2005). Relative glomerular hyperfiltration in primary aldosteronism. J Am Soc Nephrol.

[37] Nakano Y, Yoshimoto T, Fukuda T, Murakami M, Bouchi R, Minami I (2018). Effect of eplerenone on the glomerular filtration rate (GFR) in primary aldosteronism: sequential changes in the GFR during preoperative eplerenone treatment to subsequent adrenalectomy. Intern Med.

[38] Iwakura Y, Ito S, Morimoto R, Kudo M, Ono Y, Nezu M (2016). Renal resistive index predicts postoperative blood pressure outcome in primary aldosteronism. Hypertension.

[39] Ito S, Shikata K, Nangaku M, Okuda Y, Sawanobori T (2019). Efficacy and safety of esaxerenone (CS-3150) for the treatment of type 2 diabetes with microalbuminuria: a randomized, double-blind, placebo-controlled, phase II trial. Clin J Am Soc Nephrol.

