# Supplementary material for: Efficacy and safety of esaxerenone (CS-3150), a newly available nonsteroidal mineralocorticoid receptor blocker, in hypertensive patients with primary aldosteronism
Source: Hypertens Res. 2020 Nov 16;44(4):464–72. doi: 10.1038/s41440-020-00570-5 (PMC8019657; doi:10.1038/s41440-020-00570-5)
Supplement: Supplementary file 1 — Supplementary material [file 41440_2020_570_MOESM1_ESM.pdf]

Supplementary material

Efficacy and safety of esaxerenone (CS-3150), a newly available non-steroidal mineralocorticoid receptor blocker, in hypertensive patients with primary aldosteronism

Fumitoshi Satoh, Sadayoshi Ito, Hiroshi Itoh, Hiromi Rakugi, Hirotaka Shibata, Atsuhiro Ichihara, Masao Omura, Katsutoshi Takahashi, Yasuyuki Okuda, Setsuko Iijima

Supplementary table

Supplementary Table 1. Patient subgroup analysis for change in blood pressure from baseline to the end of treatment

		Patients (n)	Mean change at the end of treatment (95% CI)	
			SBP	DBP
Sex	Male	19	−13.6 (−17.5, −9.8)	−7.5 (−10.6, −4.4)
	Female	25	−20.7 (−24.9, −16.6)	−11.0 (−14.2, −7.8)
Age	<65 years	40	−17.7 (−20.8, −14.6)	−9.6 (−11.9, −7.3)
	≥65 years	4	−17.0 (−37.7, 3.7)	−8.5 (−23.3, 6.3)
Body mass index	<25 kg/m^2	23	−17.1 (−20.8, −13.4)	−8.4 (−11.1, −5.7)
	≥25 kg/m^2	21	−18.2 (−23.3, −13.1)	−10.7 (−14.5, −6.9)
PA subtype	Unilateral adrenal lesion	4	−13.0 (−21.7, −4.3)	−7.5 (−11.3, −3.7)
	Bilateral adrenal lesion	38	−17.6 (−20.9, −14.3)	−9.4 (−12.0, −6.9)
Disease classification	Aldosterone-producing tumour	4	−13.0 (−21.7, −4.3)	−7.5 (−11.3, −3.7)
	Idiopathic hyperaldosteronism	35	−18.4 (−21.8, −14.9)	−9.9 (−12.5, −7.3)

Baseline SBP	<160 mmHg	34	−17.4 (−20.5, −14.3)	−9.9 (−12.5, −7.4)
	≥160 mmHg	10	−18.6 (−27.8, −9.4)	−8.0 (−13.4, −2.6)
Baseline DBP	<100 mmHg	24	−19.2 (−23.1, −15.3)	−8.3 (−10.8, −5.9)
	≥100 mmHg	20	−15.8 (−20.6, −11.0)	−10.9 (−15.0, −6.8)
Prior use of	Yes	38	−17.5 (−20.5, −14.4)	−9.8 (−12.2, −7.3)
antihypertensive agents	No	6	−18.8 (−33.4, −4.2)	−7.8 (−15.0, −0.7)
Concomitant	Yes	30	−15.6 (−18.5, −12.8)	−8.4 (−10.4, −6.4)
antihypertensive agents	No	14	−22.0 (−29.3, −14.7)	−11.9 (−17.6, −6.1)
Baseline ARR	<median	22	−17.7 (−21.4, −14.0)	−10.7 (−13.7, −7.7)
	≥median	22	−17.6 (−22.6, −12.7)	−8.3 (−11.7, −4.9)
Comorbid type 2 diabetes	Yes	5	−15.0 (−22.2, −7.8)	−9.4 (−14.1, −4.7)
mellitus	No	39	−18.0 (−21.3, −14.7)	−9.5 (−12.0, −7.0)
PRA at the end of	≥1 ng/mL/h	17	−20.0 (−24.9, −15.1)	−10.9 (−14.6, −7.2)
treatment	<1 ng/mL/h	24	−16.5 (−20.6, −12.4)	−8.7 (−11.8, −5.6)

Values are means (95% CIs).

ARR, aldosterone–renin ratio; CI, confidence interval; DBP, diastolic blood pressure; PA, primary aldosteronism; PRA, plasma renin activity; SBP, systolic blood pressure.

Supplementary Table 2. Subgroup analysis of BP and BP change in patients with or without concomitant antihypertensive therapy

		Baseline	Week 2	Week 4	Week 8	Week 12	End of treatment
SBP, mmHg	ALL	154.0 ± 9.8	143.1 ± 12.5	138.1 ± 13.5	135.4 ± 13.0	136.7 ± 13.9	136.4 ± 13.6
	Change from baseline	0	−11.0 ± 10.3	−16.0 ± 12.9	−18.7 ± 10.6	−17.9 ± 10.1	−17.7 ± 9.8
	Without concomitant antihypertensive therapy	154.3 ± 11.0	140.4 ± 13.9	136.2 ± 14.9	129.7 ± 14.7	132.0 ± 14.9	132.3 ± 14.0
	Change from baseline	0	−13.9 ± 12.5	−18.0 ± 15.5	−24.5 ± 13.6	−22.7 ± 13.6	−22.0 ± 12.6
	With concomitant antihypertensive therapy	153.9 ± 9.4	144.4 ± 11.8	139.0 ± 13.0	137.9 ± 11.6	138.6 ± 13.2	138.3 ± 13.2
	Change from baseline	0	−9.6 ± 9.0	−15.0 ± 11.7	−16.1 ± 7.9	−15.8 ± 7.6	−15.6 ± 7.6
DBP, mmHg	ALL	100.0 ± 5.9	93.9 ± 8.8	91.1 ± 8.7	90.0 ± 9.3	90.2 ± 8.9	90.5 ± 8.6
	Change from baseline	0	−6.0 ± 7.0	−8.7 ± 7.0	−9.8 ± 8.0	−9.5 ± 7.6	−9.5 ± 7.3
	Without concomitant antihypertensive therapy	99.6 ± 5.2	91.1 ± 10.4	88.5 ± 8.6	85.5 ± 10.1	87.1 ± 10.3	87.8 ± 9.7

Change from baseline	0	−8.6 ± 9.2	−10.5 ± 7.4	−13.5 ± 9.3	−12.3 ± 10.8	−11.9 ± 10.0
With concomitant antihypertensive therapy	100.1 ± 6.2	95.3 ± 7.9	92.2 ± 8.6	92.0 ± 8.4	91.5 ± 8.1	91.7 ± 7.8
Change from baseline	0	−4.8 ± 5.4	−7.9 ± 6.8	−8.1 ± 6.8	−8.3 ± 5.5	−8.4 ± 5.4

Values are means ± standard deviations.

DBP, diastolic blood pressure; SBP, systolic blood pressure.

Supplementary figure legends

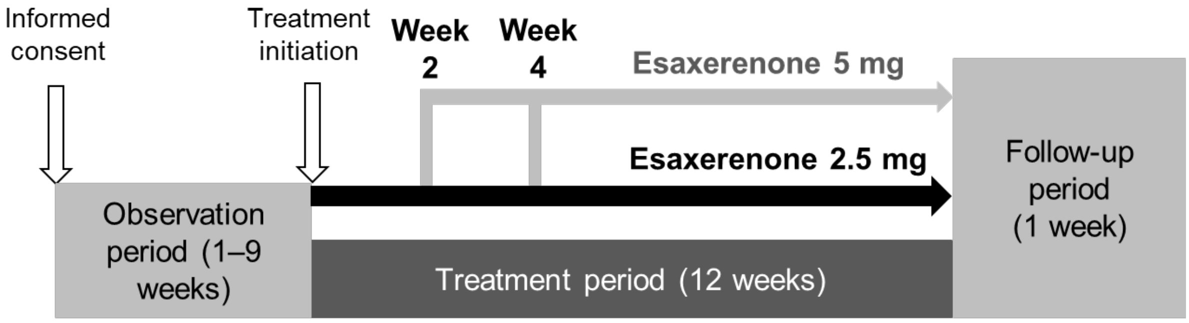

Supplementary Figure 1. Study design.

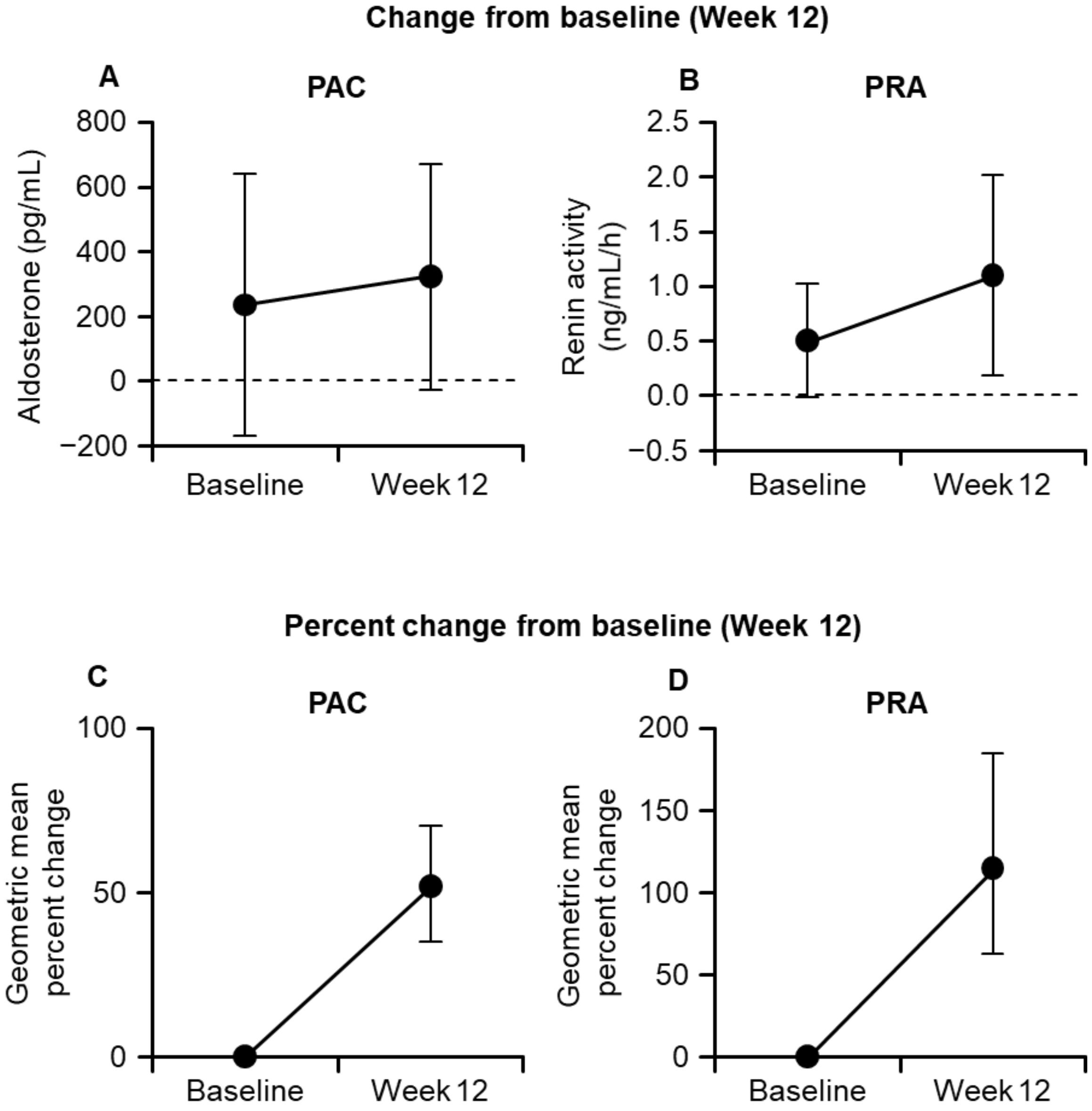

Supplementary Figure 2. Absolute change (means ± SD) from baseline in plasma aldosterone concentration (PAC) (**A**) and plasma renin activity (PRA) (**B**); and percent change from baseline in PAC (**C**) and PRA (**D**).
